# Adultes Rhabdomyom: Eine seltene Differentialdiagnose in der Schnellschnittuntersuchung

**DOI:** 10.1007/s00292-025-01443-9

**Published:** 2025-06-10

**Authors:** Marvin Seidel, Martin Bredt, Hans Kreipe, Nora Schaumann

**Affiliations:** Institute of Pathology, Hannover Medical School, Carl-Neuberg-Straße 1, 30625 Hannover, Germany

**Keywords:** Immunhistochemie, Querstreifung, Kopf-Hals-Bereich, Granularzelltumor, Rezidivrisiko, Immunohistochemistry, Cross striation, Head and neck region, Granular cell tumor, Risk of recurrence

## Abstract

Wir berichten über einen 73-jährigen Patienten mit vorbekanntem malignem Melanom und neu aufgefallenem Tumor im Larynx. In der Schnellschnittuntersuchung fand sich kein Anhalt für Malignität. Differentialdiagnostisch wurde zunächst an einen Granularzelltumor gedacht. Die Immunhistochemie und die Morphologie nach Umbettung in Paraffin erbrachten dann die abschließende Diagnose eines adulten Rhabdomyoms. Diese Kasuistik beleuchtet die histomorphologische und immunhistochemische Differentialdiagnose großzelliger Läsionen im Larynx, mit besonderem Fokus auf die Herausforderung im Schnellschnitt.

## Abstract (en)

We report on a 73-year-old patient with formerly diagnosed melanoma and newly recognized tumor formation within the larynx. In a frozen section specimen, there was no indication of malignancy. Differential diagnosis favored a granular cell tumor at first. Immunohistochemistry and histomorphology after tissue transfer into paraffin led to the diagnosis of adult rhabdomyoma. This case report examines the histomorphologic and immunhistochemic differential diagnosis of large-cell laryngeal lesions with special focus on the challenges associated with frozen section specimens.

## Anamnese

Es wird der Fall eines 73-jährigen Patienten vorgestellt. Nach Exzision eines nodulären malignen Melanoms wurde im Rahmen einer Staginguntersuchung mittels Computertomographie eine tumorsuspekte Weichteilformation gefunden. Die Anamnese ergab keine relevante Symptomatik. In der Panendoskopie zeigte sich eine unklare zystische Raumforderung der Oropharynxwand links posterior der Tonsillenloge. Aus diesem Bereich wurden Proben entnommen und im Schnellschnitt untersucht.

## Befund

**Abb. 1:**
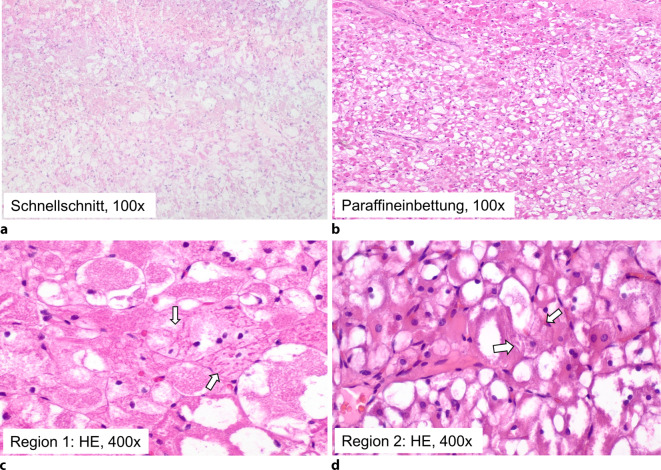
HE-Färbung Rhabdomyom. **a** Gefrierschnittaufarbeitung/Schnellschnitt, **b** Paraffineinbettung in 100facher Vergrößerung und in (**c**–**d**) 400facher Vergrößerung mit intrazytoplasmatischen fadenförmigen Einschlüssen (*Pfeile*)

Mikroskopisch fand sich randständig unverhornte plattenepitheliale Schleimhaut mit regelhafter basoapikaler Ausreifung, ohne Atypien oder Ulzeration. Subepithelial präsentierte sich mit scharfer Begrenzung ein monomorphes Proliferat aus großen polygonalen Zellen mit scharfen Zellgrenzen, mit teils eosinophilem, teils granulärem und teils hellzelligem Zytoplasma sowie mit überwiegend zentralen oder parazentralen kleinen Zellkernen mit unauffälliger, homogener Chromatinstruktur und teils kleinen Nukleolen. Mitosen, Nekrosen oder nukleäre Atypien waren nicht auszumachen (Abb. 1a). Als Schnellschnittdiagnose wurde ein benignes Proliferat favorisiert, in Zusammenschau der Morphologie und der Lokalisation im Oropharynx in erster Linie ein Granularzelltumor. Anhalt für Malignität, insbesondere für eine Absiedlung des vorbekannten malignen Melanoms, bestand nicht. Die Schnellschnitte wurden anschließend routinemäßig in Formalin fixiert und in Paraffin eingebettet.

Hier zeigte sich ein zum Schnellschnitt morphologisch ähnliches, nicht bekapseltes Proliferat neben Plattenepithel und ortsständigem lymphatischem Gewebe. Das Proliferat erschien glykogenreich (PAS-positiv, Diastase-negativ). Der Befund wies keine histomorphologischen Merkmale eines malignen Melanoms auf. Prinzipiell war das Bild morphologisch mit dem zunächst favorisierten Granularzelltumor zu vereinbaren.

**Abb. 2:**
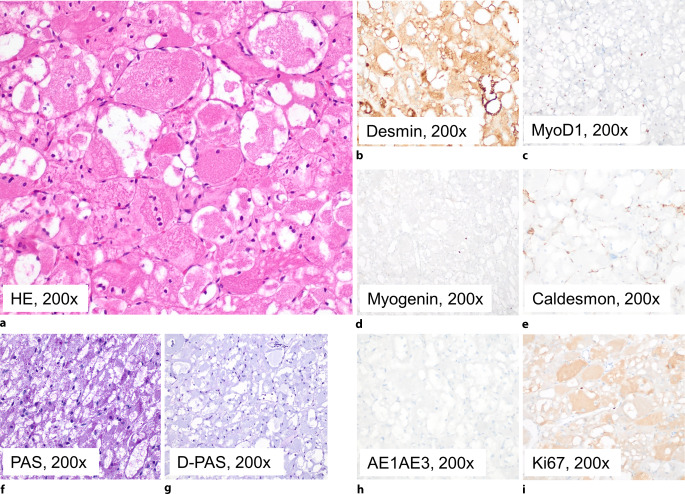
Histochemische und immunhistochemische Färbung des Rhabdomyoms. **a** HE, **b** Desmin positiv, **c** MyoD1 positiv (nukleär), **d** Myogenin negativ, **e** Caldesmon negativ, **f** PAS positiv, **g** PAS-Diastase negativ, **h** AE1/AE3 negativ, **i** Ki67 mit sehr geringer Proliferation. Vergrößerung jeweils 200:1

In der immunhistochemischen Färbereaktion präsentierte sich das Proliferat negativ für Panzytokeratin, S100, CD68 (PGM-1), Melan‑A, HMB-45 und SOX-10, was ein malignes Melanom sowie einen Granularzelltumor oder ein Hibernom diagnostisch unwahrscheinlich erscheinen ließ. Der Proliferationsindex Ki67 lag bei < 1 %. Bezüglich einer myogenen Differenzierung zeigte sich eine kräftige Expression von Desmin sowie partiell für skelettmuskelspezifisches Aktin (SMA) und für Myo-D1. Färbungen gegen Myogenin und Caldesmon fielen negativ aus (Abb. 2).

Rückblickend fanden sich in der Hämatoxylin-Eosin-Färbung fokal intrazytoplasmatische fadenförmige, oftmals gruppierte Einschlüsse (Abb. 1b–d), welche in der Schnellschnittuntersuchung nicht sichtbar waren. In Kenntnis der immunhistochemischen Expression rhabdomyogener Marker waren die intrazytoplasmatischen fadenförmigen Einschlüsse als vereinzelte Residuen einer Querstreifung einzuordnen. In Zusammenschau der Befunde ergab sich die endgültige Diagnose eines adulten Rhabdomyoms.

Ein fetales Rhabdomyom lag aufgrund morphologischer Kriterien nicht vor. Es fanden sich Zellen, welche an reife Rhabdomyozyten erinnern. Diese wiesen eine polygonale Form und breites Zytoplasma auf. Merkmale eines fetalen Rhabdomyoms wie unreife Rhabdomyoblasten, eine myxoide Grundsubstanz oder primitive Rund- oder Spindelzellen stellten sich nicht dar.

## Diagnose und Verlauf

Der Patient wurde auf der interdisziplinären Tumorkonferenz vorgestellt. Im Fokus stand dabei die adjuvante Therapie des malignen Melanoms. Das adulte Rhabdomyom erforderte bei vollständiger Resektion keine weitere Therapie.

## Diskussion

Als adultes Rhabdomyom wird ein seltener, benigner Weichteiltumor bezeichnet, der eine Differenzierung der quergestreiften Muskulatur zeigt [3]. Es wird unterschieden zwischen den häufigeren kardialen und den selteneren extrakardialen Rhabdomyomen. Das extrakardiale Rhabdomyom wird in die Subtypen adult, fetal und genital unterschieden [2]. Die adulte Form des Rhabdomyoms wird im Durchschnitt im Alter von 65 Jahren diagnostiziert und betrifft häufiger Männer als Frauen (4:1) [5]. Die Ätiologie des Rhabdomyoms ist unbekannt [2].

Eine Prädilektionsstelle ist der Kopf-Hals-Bereich (90 % aller adulten Rhabdomyome). Konkret im Larynx können die Tumoren auch multizentrisch vorkommen, dabei asymptomatisch bleiben oder insbesondere durch eine Dysphonie oder Dysphagie auffallen [4–6].

Bei kardialer Manifestation von Rhabdomyomen ist eine Assoziation mit tuberöser Sklerose beschrieben (mit Alterationen im *TSC‑1*[9q34]- oder *TSC‑2*[16q13]-Gen) [7]. Für extrakardiale adulte Rhabdomyome hingegen ist eine Assoziation mit tuberöser Sklerose nicht eindeutig beschrieben [4, 5].

Der hier vorliegende Fall eines extrakardialen adulten Rhabdomyoms ist in seiner Präsentation typisch. Als morphologisches Merkmal des Rhabdomyoms steht vor allem die residuelle Querstreifung im Fokus [4]. Diese feinen intrazytoplasmatischen Strukturen bleiben aufgrund des Gefrierschnittverfahrens in der Schnellschnittuntersuchung nicht erhalten. Damit ist im Schnellschnitt die typische Morphologie maskiert. Differentialdiagnostisch wurde aufgrund der Anamnese an eine Absiedlung eines malignen Melanoms gedacht. Des Weiteren kamen insbesondere auch andere benigne Tumoren im Kopf-Hals-Bereich in Betracht, wie beispielsweise ein Granularzelltumor. Der Granularzelltumor ist eine seltene, aber bekannte Fehldiagnose in der Gefrierschnittuntersuchung bei Rhabdomyomen im Larynx [5].

Unter Zuhilfenahme der Immunhistochemie sind Rhabdomyome (S100^–^, SOX-10^–^, CD68^–^) und Granularzelltumoren (S100^+^, SOX-10^+^, CD68^+^, myogene Marker negativ) gut zu differenzieren [2]. Adulte Rhabdomyome exprimieren immunhistochemisch (rhabdo-)myogene Marker. Insbesondere Desmin ist typischerweise diffus positiv. Auch MyoD1 ist typischerweise positiv. Myogenin hingegen kann negativ ausfallen oder nur eine fokale Positivität aufweisen und ist daher in der Diagnostik weniger zuverlässig [8]. Weitere Differentialdiagnosen können Onkozytome oder Paragangliome umfassen [4].

Es wird bei kleinen asymptomatischen Tumoren eine beobachtende Haltung und bei großen symptomatischen Tumoren eine Exzision in toto angestrebt [1, 5, 6]. Auch wenn vom adulten Rhabdomyom kein Entartungsrisiko ausgeht, besteht ein Rezidivrisiko. Ein Rezidiv wird insbesondere durch eine inkomplette Entfernung begünstigt [1, 4, 6]. Klinische Kontrollen sind deshalb postoperativ anzuraten.

## Fazit für die Praxis

- Adulte Rhabdomyome sind eine Rarität und können insbesondere in der Gefrierschnitttechnik eine Herausforderung darstellen, wenn die typische Morphologie nicht erhalten bleibt.
- Eine immunhistochemische Aufarbeitung sowie die Darstellung der residuellen Querstreifung kann die Diagnose sichern.
- Es handelt sich um einen benignen Tumor. Aufgrund der Rezidivgefahr sind klinische Kontrollen relevant.
